# 3D/4D Printing Application for Shape Memory Materials

**DOI:** 10.3390/ma15175999

**Published:** 2022-08-30

**Authors:** Andrea Ehrmann

**Affiliations:** Faculty of Engineering and Mathematics, Bielefeld University of Applied Sciences, 33619 Bielefeld, Germany; andrea.ehrmann@fh-bielefeld.de

This new Special Issue of Materials entitled “*3D/4D Printing Application for Shape Memory Materials*” aims to publish original and review papers dealing with basic and applied research on this emerging technology. Three-dimensional printing offers a considerable amount of freedom in design, as well as the possibility to create individualized objects, e.g., for medical or protection purposes. Several 3D printing techniques, e.g., fused deposition modeling (FDM) or stereolithography (SLA), are currently available for diverse applications.

While 3D printing has steadily developed further toward higher precision, faster printing, and lower costs, the so-called 4D printing is much younger. In a few words, 4D printing describes 3D-printed objects that can afterward change their shapes upon triggers such as heat or light [1]. This effect can, on the one hand, be reached by combining different materials, such as elastic printed polymer on stretchable textile fabric [2]. On the other hand, some 3D printing materials, such as poly(lactic acid) (PLA), which is often used in fused deposition modeling, show an additional shape memory effect, meaning that the material “remembers” its original shape after deformation, thus being able to recover its original form due to an external stimulus, e.g., heat [3]. Such changes in the shape of an object with time can occur once or even bidirectionally. Generally speaking, 4D-printed objects can be used for a broad range of applications, from design to functional properties, and from medical purposes to soft robotics.

This Special Issue aims to amass recent material innovations and applications of 3D-printed shape memory materials for shape recovery and 4D printing. Recent experimental and theoretical studies are as welcome as comprehensive reviews regarding these topics.
